# Left ventricular false tendons

**DOI:** 10.1007/s12471-021-01592-5

**Published:** 2021-07-07

**Authors:** S. Velthuis, P. J. Senden

**Affiliations:** Department of Cardiology, Meander Medical Centre, Amersfoort, The Netherlands

**Keywords:** Left ventricular false tendon, Ventricular arrhythmias, Abnormal cardiac remodelling

## Abstract

Left ventricular false tendons (LVFTs) are fibromuscular structures, connecting the left ventricular free wall or papillary muscle and the ventricular septum.

There is some discussion about safety issues during intense exercise in athletes with LVFTs, as these bands have been associated with ventricular arrhythmias and abnormal cardiac remodelling. However, presence of LVFTs appears to be much more common than previously noted as imaging techniques have improved and the association between LVFTs and abnormal remodelling could very well be explained by better visibility in a dilated left ventricular lumen.

Although LVFTs may result in electrocardiographic abnormalities and could form a substrate for ventricular arrhythmias, it should be considered as a normal anatomic variant. Persons with LVFTs do not appear to have increased risk for ventricular arrhythmias or sudden cardiac death.

## Case

**Fig. 1:**
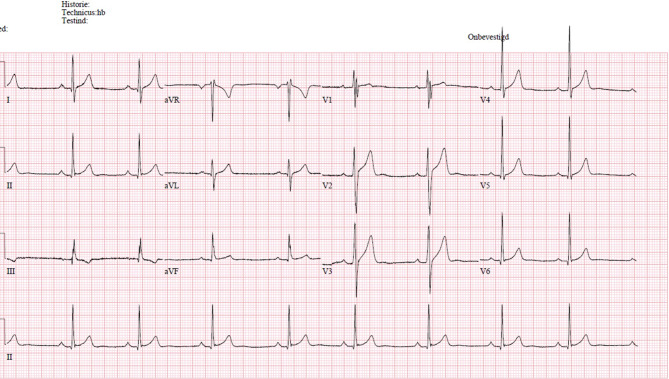
Electrocardiogram with fragmented QRS complex in leads III and V1

**Fig. 2:**
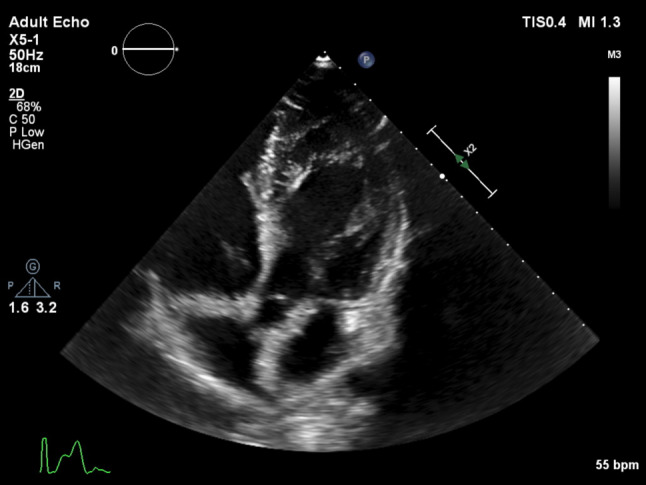
Transthoracic echocardiogram. Apical 5‑chamber view demonstrating prominent left ventricular false tendon from the septal base to the apex

An 18-year-old elite soccer player was seen at our outpatient department for routine sports cardiovascular screening. He had no cardiac symptoms, palpitations, near collapse or family history of sudden death. With physical examination no abnormalities were present. His electrocardiogram revealed a fragmented QRS complex in leads III and V1 (Fig. 1). QRS width was 100 msec. Cardiopulmonary stress testing revealed no abnormalities, specifically no premature ventricular contractions or rhythm disorder. A prominent left ventricular false tendon from the septal base to the apex was present at the echocardiogram (Fig. 2). Additional cardiac magnetic resonance imaging (MRI) excluded other structural abnormalities, such as non-compaction cardiomyopathy, and showed no late gadolinium enhancement. Since there is some discussion about safety issues during intense exercise in elite athletes with false tendons, we reviewed the literature and performed a systematic search of peer-reviewed studies that examined the clinical significance of left ventricular false tendons. Literature was searched using the PubMed database up to December 2020 using the keywords false tendon or false chordae tendineae. Each reference list was screened additionally.

## Introduction

Left ventricular false tendons (LVFTs) are echogenic fibromuscular structures, connecting the left ventricular free wall or papillary muscle and the ventricular septum. As they are not related to the mitral valve apparatus, the term “false” tendon is in use. They should be differentiated from other entities such as thickened ventricular trabeculations or ventricular masses (Tab. 1). Especially diagnosis of left ventricular non-compaction can be challenging and LVFTs should not be mistaken for atypical variants of this form of cardiomyopathy [1, 2].

**Table 1:** Differential diagnosis

*Diagnosis*	*Description*
False tendons	Stringlike structures with free intracavitary course and connected to ventricular walls and/or papillary muscles. Fibrous, muscular or fibro-muscular composition
Trabeculation	Localised protrusions of the ventricular wall that have no free intracavitary course
Hypertrabeculation	> 3 trabeculations associated with intraventricular recesses
Non-compaction cardiomyopathy	Congenital primary cardiomyopathy characterised by sponge-like appearance of left ventricular myocardium
Intra-cardiac thrombus or tumour	Intra-cardiac thrombi or tumours are seen in a variety of clinical settings and can result in severe morbidity from embolic events

Based on their location in the apical-, mid-, or basal-third segment along the long axis of the left ventricle, LVFTs can be classified as transverse (localised to one zone), diagonal (across two zones) or longitudinal (across all three zones). Most LVFTs are transverse and located in the apex [3], although LVFTs connecting the basal ventricular septum to the apical segment of the lateral free wall are also a frequent finding [4]. LVFTs can be simple, with a cord traversing the left ventricle with 1–2 points of insertion, or complex with 3 or more insertion points (branching).

## Prevalence of LVFTs

The exact prevalence of LVFTs in the general population is unclear. Different studies report a widely varying prevalence between 0.4% to 83% [4, 5]. The detection rate can be low, as LVFTs may be ignored by echocardiographers or require additional planes to be detected. However, increased level of attention for LVFTs and the development of echocardiographic technologies and cardiac MRI have increased the detection rate of LVFTs. The only available community-based, prospective study is performed by Kenchaiah et al. and part of the well-known Framingham Heart Study [3]. This analysis between 1987 and 1990 demonstrated presence of an LVFT in 101 of 3931 (2.6%) eligible persons screened. However, given the progress in imaging techniques during the recent years, this low percentage is surely an underestimate of the real prevalence of LVFTs. In fact, LVFTs were also found in 32 out of 176 (18%) age and sex matched persons, where an LVFT was unidentified during initial screening of this cohort. This phenomenon is also seen in a study by Hall et al., where 15 out of 100 matched “controls” appeared to have an LVFT, supporting a more frequent prevalence of LVFTs in the general population [6]. An autopsy study by Luetmer et al. described presence of LVFTs in 55% of hearts [7] and LVFTs were present in 78% and 83% of hospital-referred children and healthy subjects respectively [4]. The reported prevalence of LVFTs can also be subject to selection bias, as presence of LVFTs have been associated with innocent precordial murmurs. Furthermore, it is possible that LVFTs are more commonly found in young non-obese persons and in patients with dilated left ventricles, since these factors increase their visibility on echocardiography.

## Clinical significance of LVFTs

Although LVFTs are generally considered as benign anatomic variants, they have been associated with ventricular arrhythmias [8] and abnormal cardiac remodelling, including systolic and diastolic dysfunction and dilatation [6].

### LVFTs and cardiac remodelling

Hall et al. retrospectively evaluated echocardiographic and clinical parameters of 126 patients with identified LVFTs and compared them with 85 age-matched controls without LVFTs [6]. In this study, patients with LVFTs had more prevalent heart failure, more left ventricular dilatation, were more likely to have moderate to severe mitral regurgitation and had more severe systolic and diastolic dysfunction. An LVFT location near the middle and basal left ventricular segments was associated with increased left ventricular dysfunction and dilatation, while this relation was not significant for apical LVFTs. This study suggests that LVFTs may be associated with adverse structural and functional left ventricular changes, although the mechanism by which LVFTs may cause reduced systolic and diastolic function and increased dilatation is unclear. Because the study by Hall et al. was performed in a tertiary referral centre with a cross-sectional design, a clear cause-effect relationship cannot be determined and the pathophysiologic mechanism is unknown. There has also been a selection bias in this study, as heart failure was present in up to 21% of control patients without LVFTs. The association between LVFTs and left ventricular dilatation could very well be explained by better visibility in a dilated left ventricular lumen.

Interestingly, a similar structure in the right ventricle is named the moderator band, as it was thought that its attachment instead prevents overdistension of this ventricle [9] and it has been suggested that LVFTs may retard cardiac remodelling by tethering the left ventricular wall to which they are attached. Furthermore, LVFTs that run between the papillary muscles may stabilise these structures and retard the occurrence of functional mitral valve regurgitation.

### LVFTs and ECG

Presence of LVFTs may be associated with abnormal ventricular repolarisation in young healthy subjects in competitive sports [4, 10–12]. Inverted symmetrical, sometimes biphasic, T waves can be seen (especially in V1–V3) which regress with increase in heart rate during exercise testing. Furthermore, the presence of LVFTs has been related to the presence of J waves, so-called terminal QRS notching or slurring [10].

### LVFTs and ventricular arrhythmias

Small case series suggest an increased risk of ventricular tachycardia [8, 13–15] and speculate that conduction through LVFTs containing Purkinje fibres constitute part of the tachycardia network or that an LVFT may produce stretch in the interventricular septum with increased automaticity. Ventricular premature beats and/or arrhythmias might then be triggered due to increased automaticity of these conducting cells during mechanical stretch of the left ventricular wall at the insertion point. Thakur et al. describe a case series of 15 patients referred for catheter ablation of idiopathic left ventricular tachycardia (ILVT) [8]. This type of tachycardia is characterised by QRS complexes with a right bundle branch block morphology and left axis deviation and can be seen in patients without structural heart disease. It is often precipitated during exercise and usually responds to intravenous verapamil. In this study by Thakur et al., LVFT was found in all patients referred for ILVT ablation, while LVFT was found in 5% of the control group. Radiofrequency catheter ablation of the postero-apical septum resulted in cure in 14 of 15 patients. This suggests that the LVFT may be responsible for this type of tachycardia. The mechanism by which the LVFT precipitates this tachycardia remains speculative. One possible explanation might be that conduction through the LVFT constitutes part of the ventricular tachycardia circuit. Or maybe the tendon produces stretch in the Purkinje fibre network in the interventricular septum. Just as the papillary muscles and right ventricular moderator band, conduction tissue in LVFTs can be the source of ventricular arrhythmias [9].

### LVFTs and mortality

In the only prospective community-based Framingham study with a mean follow-up of 7.7 years, 15 of 101 (15%) individuals with LVFTs and 19 of 151 (13%) participants without LVFTs died. In multivariable models, presence of LVFTs was not associated with the risk of mortality [3].

## Conclusion

LVFTs are a frequent finding in the general population, especially in young, non-obese persons. Just like other bands in the heart, LVFTs seem to be a normal anatomical structure. LVFTs may participate in electrocardiographic conduction and can theoretically be the source of ventricular arrhythmias. At this moment, there is no solid evidence relating LVFTs to excess cardiac morbidity and mortality and it would not be appropriate to classify persons with LVFTs as persons who have an increased risk of ventricular arrhythmias or sudden cardiac death.
